# Flow-injection sandwich ELISA for bioprocess monitoring

**DOI:** 10.1155/S1463924699000140

**Published:** 1999

**Authors:** Jong Il Rhee, Jörg Hagedorn, Thomas Scheper, Karl Schügerl

**Affiliations:** 1 Institut für Technische Chemie Universität Hannover Callinstrasse 3 Hannover D-30167 Germany; 2 Department of Biochemical Engineering Chonnam National University YongBong-Dong 300 Kwangju 500-757 Korea

## Abstract

A fully automated flow-injection immunoassay based on sandwich enzyme-linked immunosorbent assay (ELISA) is described for the model system: protein G-sepharose, rabbit IgG and horseradish peroxidase (HRP)-labelled protein A. After injecting rabbit IgG and HRP-labelled protein A into a cartridge containing protein G-sepharose sequentially, a mixture of hydrogen peroxide and the redox indicator, 2.2′-azino-bis(3-ethylbenzthiazoline-6-sulphonic acid) (ABTS) is passed through the cartridge. The HRP-labelled protein A bound in the cartridge is directly proportional to the concentration of rabbit IgG. The colour variation of ABTS caused during the reaction between HRP and H_2_0_2_ in the cartridge is detected photometrically. The whole assay procedure is controlled and evaluated by a computer. Rabbit IgG and HRP-labelled protein A are also detected by a fluorometer, which is introduced into the flow system. In the flow-injection sandwich ELISA, the slope of the calibration curve is 0.4491 in the range of 0 and 300 μg ml^-1^ rabbit IgG, while it is 0.1274 in the heterogeneous immunoassay. So the flow-injection sandwich ELISA system is found to be more sensitive than a heterogeneous immunoassay for the monitoring of the model protein.


Journal of Automated Methods & Management in Chemistry, Vol. 21, No. 4 (July–August 1999) pp. 121–125

### Flow-injection sandwich ELISA for bioprocess monitoring

**Jong Il Rhee,[1] Jörg Hagedorn, Thomas Scheper and Karl Schügerl**

*Institut für Technische Chemie, Universität Hannover, Callinstrasse 3, D-30167 Hannover, Germany; [1]Department of Biochemical Engineering, Chonnam National University, YongBong-Dong 300, 500-757 Kwangju, Korea*

*A fully automated flow-injection immunoassay based on sandwich enzyme-linked immunosorbent assay (ELISA) is described for the model system: protein G-sepharose, rabbit IgG and horseradish peroxidase (HRP)-labelled protein A. After injecting rabbit IgG and HRP-labelled protein A into a cartridge containing protein G-sepharose sequentially, a mixture of hydrogen peroxide and the redox indicator, 2.2′-azino-bis(3-ethylbenzthiazoline-6-sulphonic acid) (ABTS) is passed through the cartridge. The HRP-labelled protein A bound in the cartridge is directly proportional to the concentration of rabbit IgG. The colour variation of ABTS caused during the reaction between HRP and $H_2O_2$ in the cartridge is detected photometrically. The whole assay procedure is controlled and evaluated by a computer. Rabbit IgG and HRP-labelled protein A are also detected by a fluorometer, which is introduced into the flow system. In the flow-injection sandwich ELISA, the slope of the calibration curve is 0.4491 in the range of 0 and 300 $\mu g\ ml^{-1}$ rabbit IgG, while it is 0.1274 in the heterogeneous immunoassay. So the flow-injection sandwich ELISA system is found to be more sensitive than a heterogeneous immunoassay for the monitoring of the model protein.*

### Introduction

On-line monitoring of biotechnological processes is important for a better understanding of cell growth and for process improvements. In mammalian cell cultivation processes, the microtitre-plate enzyme-linked immunosorbent assay (ELISA) is normally used to measure concentrations of protein products. However, this ELISA technique is time consuming and labour intensive.

Some efforts have been made to combine the conventional ELISA with flow-injection analysis (FIA) for the on-line monitoring of protein products [1–3]. De Alwes and Wilson reported a concept for both sandwich- and competitive-type assays with glucose oxidase [4, 5]. Based on the competitive binding between the enzyme-labelled antibody and antigen, Lee and Meyerhoff presented a non-equilibrium flow-injection ELISA system using an immobilized secondary-antibody reactor and an ammonium ion-selective potentiometric detector [6]. Recently, Nilsson and co-workers described a competitive flow ELISA system using a spectrophotometer for the monitoring of IgG [7] and $\alpha$-amylase [8]. However, there are no systematic studies on a flow-injection sandwich ELISA using fluorescence detection for the monitoring of protein products.

This paper describes a flow-injection sandwich ELISA system using fluorescence detection, the principle of which is illustrated in figure 1. Antibodies are immobilized covalently on a support and integrated in a cartridge, which is placed in the flow-injection system (A). After injection of the sample containing antigen (B), the cartridge is washed and enzyme-labelled antibody is passed through the cartridge, which binds to the antigen (C). In a rinsing step, unbound enzyme-labelled antibody is washed out from the cartridge by the carrier buffer solution. After substrate injection, a reaction between substrate and bound enzyme-labelled antibody occurs (D). The extent of the reaction is proportional to the concentration of antigen in the sample, as measured via a photometric detector. Afterwards, both bound antigen and enzyme-labelled antibody are eluted for the next assay (E).

In our work, protein G-sepharose is utilized as support material, rabbit IgG as model analyte and HRP-labelled protein A as conjugate. For the enzymatic reaction with bound HRP-labelled protein A, hydrogen peroxide ($H_2O_2$) is chosen as substrate and ABTS as the redox indicator, so the colour variation of the reaction is detected photometrically. Effects of elution buffer, carrier flow stop time and binding capacity are investigated for the model system. The sensitivity of the flow-injection sandwich ELISA system is also compared with that of a heterogeneous immuno-FIA system [9].

### Experimental

### *Apparatus*

Figure 2 shows a schematic set-up of a flow-injection sandwich ELISA system. Two six-way selection valves are employed. One is used to select sample and conjugate for the sample loop (S1), the other is used to switch substrate and buffer flows (S2). A cartridge containing 1 ml protein G-sepharose is placed behind an injection valve with 125 µl injection loop volume. The enzymatic reaction of the conjugate with substrate in the cartridge is detected by a photometer (Skalar, Model 6010) with a 500 µl micro flow-through cell (Hellma). For the detection of proteins during the elution step, a spectrofluorometer with a 40 µl flow-through cell (bandwidth of 15 nm, output of 1 V, Merck-Hitachi) is introduced into the flow system and connected to a computer. Tubes with 0.5 mm i.d. are used to connect valves and detectors. The lengths of tubing connections are as follows: 40 cm between selection valve 1 (S1) and injector, 25 cm between selection valve 2 (S2) and injector, 10 cm between injector and cartridge, 25 cm between cartridge and a micro flow-cell of the photometer, 12 cm between the micro flow-cell of the photometer and a micro cell of the fluorometer. All valves and pumps in the flow-ELISA system are controlled by a computer using the software

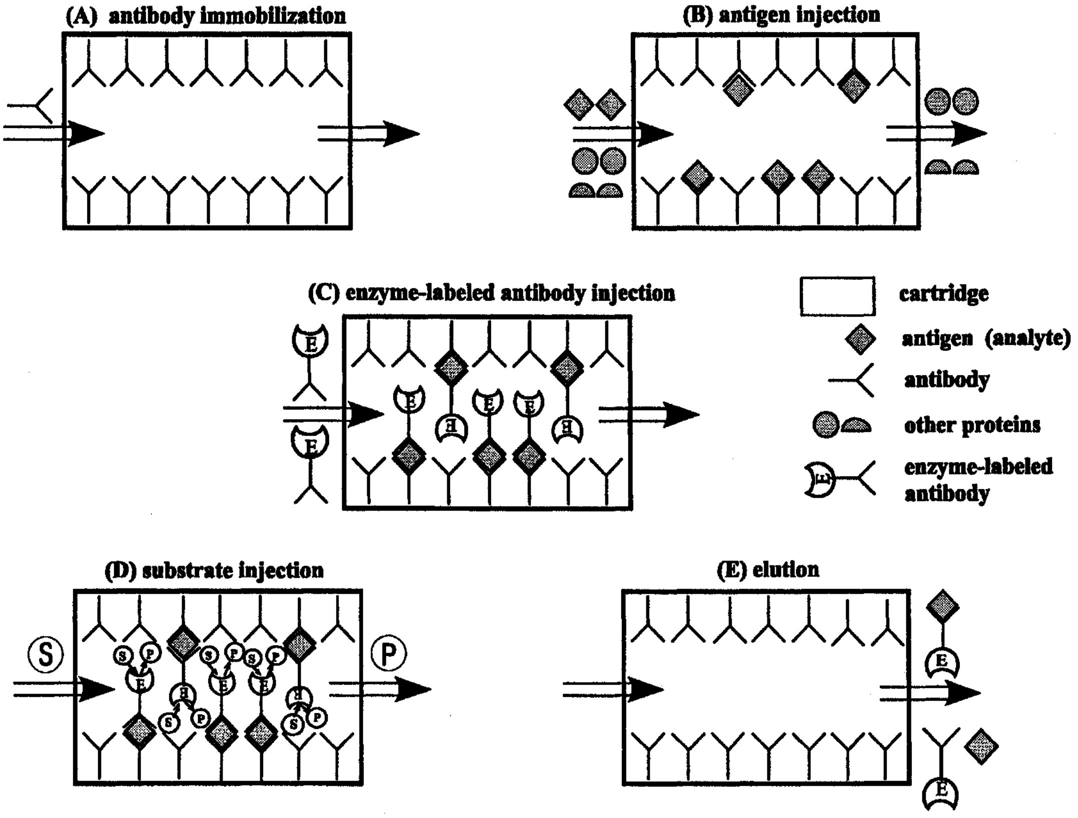

*Figure 1. Principle of a flow-injection sandwich ELISA.*

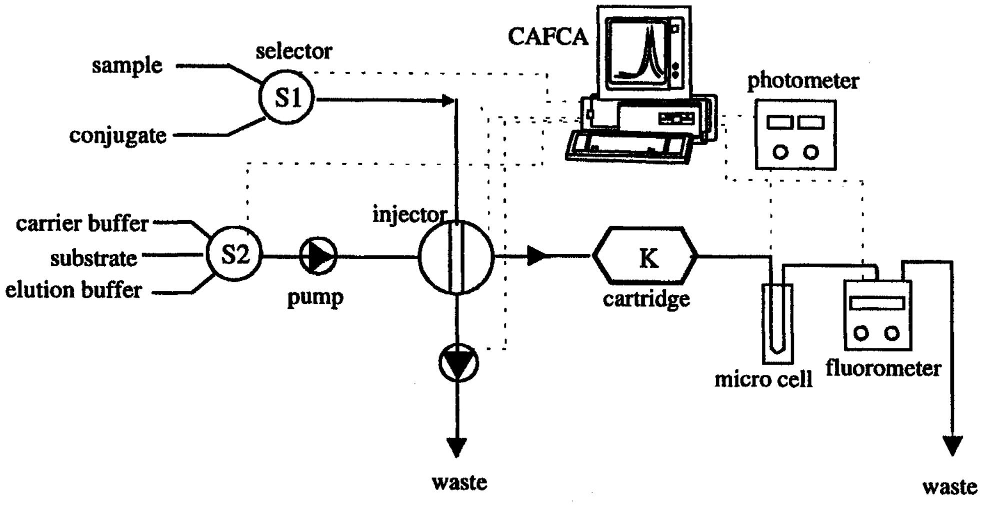

*Figure 2. Schematic set-up of a flow-injection sandwich ELISA system.*

CAFCA (computer-assisted flow and control analysis, ANASYSCON Co.). This software is also used for measurement and evaluation of the signals.

### *Reagents*

The following reagents were used: protein G-sepharose 4B fast flow (Pharmacia), horseradish peroxidase (HRP)-labelled protein A (Sigma), 2.2′-azino-bis(3-ethylbenzthiazoline-6-sulphonic acid) (ABTS, Sigma), hydrogen peroxide (Sigma), rabbit IgG (Sigma), human IgG (Sigma) and anti-A Mab (Boehringer Ingelheim Pharma KG.). Potassium phosphate buffer (0.1 M, PPB, pH 7.4, Fluka) is used as carrier buffer and glycine–HCl (0.1 M, pH 2.0, Fluka) as elution buffer. The substrate solution is prepared by mixing 0.3 g $l^{-1}$ $H_2O_2$ with 1.333 g $l^{-1}$ ABTS dissolved in deionized water, and kept at 4 °C in a dark bottle prior to use.

### *Flow-injection sandwich ELISA*

As shown in figure 2, the cartridge containing protein G-sepharose is washed continuously with carrier buffer at a flow rate of 0.8 ml $min^{-1}$. Rabbit IgG as sample is injected into the flow system and binds to protein G in the cartridge. After washing out unbound rabbit IgG in the cartridge with carrier buffer, HRP-labelled protein A is injected as conjugate and then binds to rabbit IgG in the cartridge. After washing the cartridge with carrier buffer, substrate is injected through the second selector into the cartridge for 40 s. The reaction between HRP and substrate takes place in the cartridge. The redox indicator, ABTS, is catalysed by $H_2O_2$ in the presence of HRP according to the reaction:

$$ABTS_{red} + H_2O_2 \xrightarrow{HRP} ABTS_{ox} + H_2O$$

The green colour of $ABTS_{ox}$ is detected at 425 nm by a photometer [10]. To dissociate the interaction between rabbit IgG and protein G, elution buffer was used. During the elution step, rabbit IgG and HRP-labelled protein A eluted were detected by a fluorometer at the excitation wavelength of 280 nm and the emission wavelength of 330 nm. The cartridge containing protein G-sepharose was then reequilibrated by carrier buffer for a new assay. The total assay time used in this study was 20 min, summarized as follows. Washing of cartridge (80 s); sample injection (40 s); washing of cartridge (140 s); conjugate injection (40 s); washing of cartridge (140 s); substrate injection (40 s); washing of cartridge (300 s); elution of bound rabbit IgG and HRP-labelled protein A (240 s); reequilibration of cartridge (180 s).

### Results and discussion

### *Assay cycle*

Figure 3 shows a typical timing sequence, and resulting photometric and fluorometric output for 50 μg $ml^{-1}$ and 100 μg $ml^{-1}$ rabbit IgG. For this assay, a 1:400 dilution (0.2 U HRP $ml^{-1}$) of HRP-labelled protein A is used as conjugate. First, two small peaks were detected by the fluorometer, resulting from unbound rabbit IgG (Fp1) and HRP-labelled protein A (Fp2). After substrate solution is introduced into the cartridge, a reaction between bound HRP-labelled protein A and substrate takes place. The colour variation of the redox indicator (ABTS) caused by the reaction is detected by the photometer. The peak height measured is proportional to the concentration of rabbit IgG. During the elution step, both bound rabbit IgG and HRP-labelled protein A are eluted from the cartridge and produce a third peak (Fp3) which is detected fluorometrically.

Enzyme-labelled antibody, i.e. conjugate, should be normally introduced into a sandwich ELISA in excess to analyte. In this work, HRP-labelled protein A binds to IgG, so that protein A should be introduced into the flow-ELISA in excess to IgG. However, the concentration of protein A in HRP-labelled protein A used is not given. Therefore, unbound HRP-labelled protein A should be detected fluorometrically in order to investigate whether HRP-labelled protein A diluted was introduced in excess to IgG. As shown in figure 3, there were fluorometric outputs for unbound HRP-labelled protein A, when a 1:400 dilution (0.2 U HRP $ml^{-1}$) of HRP-labelled protein A was used as conjugate. From this result

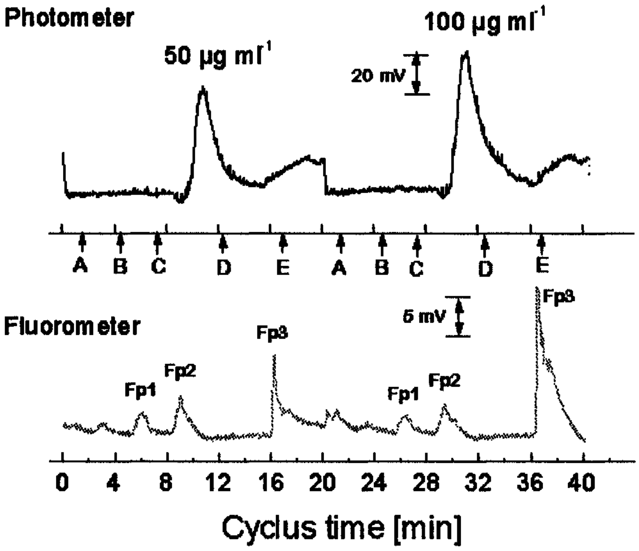

*Figure 3. Typical assay cycle in the flow-injecton sandwich ELISA system for 50 and 100 μg $ml^{-1}$ with photometric and fluorometric detection (A, sample injection; B, conjugate injection; C, substrate injection; D, elution; E, reequilibration).*

it can be said that a 1:400 dilution (0.2 U HRP $ml^{-1}$) of HRP-labelled protein A can be employed for the measurement of 100 μg $ml^{-1}$ rabbit IgG. There was also a fluorometric output of unbound HRP-labelled protein A, when a 1:500 dilution (0.16 U HRP $ml^{-1}$) of HRP-labelled protein A is used for the measurement of 50 μg $ml^{-1}$ rabbit IgG.

### *Effects of elution buffer*

The regeneration extent of the cartridge plays an important role for repeated use of the cartridge containing protein G-sepharose. The influence of elution buffer on the dissociation of binding between rabbit IgG and protein G is investigated for 0 and 100 μg $ml^{-1}$ rabbit IgG. Under the conditions outlined in the flow-injection sandwich ELISA, 100 μg $ml^{-1}$ rabbit IgG was first measured using a 1:100 dilution (0.8 U HRP $ml^{-1}$) of HRP-labelled protein A as conjugate. After the measurement the cartridge is eluted with an elution buffer. Next, a sample containing no rabbit IgG is injected, then conjugate and substrate serially. Figure 4 shows the peak heights of 0 and 100 μg $ml^{-1}$ rabbit IgG with four different elution buffers normally used. The largest difference of peak heights is obtained with elution buffer A (0.1 M glycine–HCl at pH 2.0). Even if the difference with elution buffer C (3 M NaSCN) is larger than that with elution buffer B (0.1 M $K_3PO_4$ at pH 12.3), it has been shown that there was noise phenomenon in the signal with elution buffer C. The dissociation of binding between protein G and rabbit IgG did not occur with elution buffer D (4 M urea). The peak height of 0 μg $ml^{-1}$ rabbit IgG was a little higher than that of 100 μg $ml^{-1}$ rabbit IgG, because a little HRP-labelled protein A injected during the measurement of 0 μg $ml^{-1}$ rabbit IgG was accumulated in the cartridge. From these results, 0.1 M glycine solution at pH 2.0 is found to be a good elution buffer for the dissociation of the binding

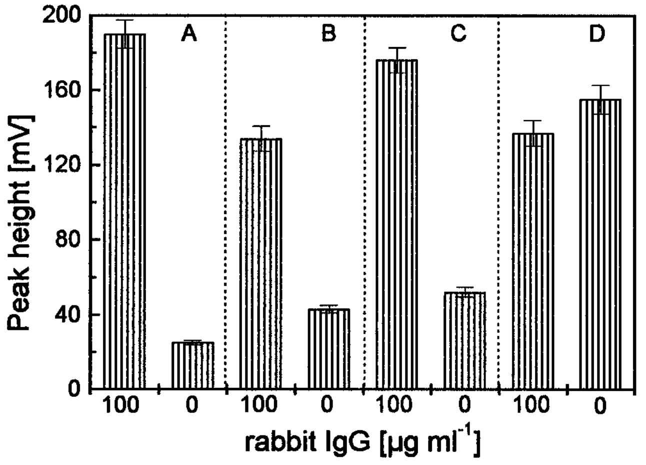

*Figure 4. Peak heights for 0 and 100 μg ml$^{-1}$ rabbit IgG with different elution buffers (A, 0.1 M glycine–HCl at pH 2.0; B, 0.1 M $K_3PO_4$ at pH 12.3; C, 3 M NaSCN; D, 4 M urea).*

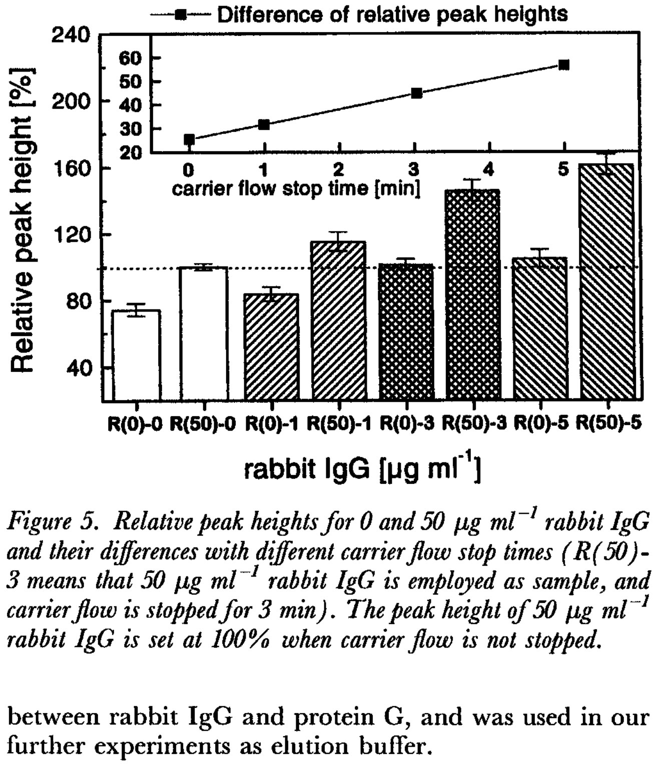

*Figure 5. Relative peak heights for 0 and 50 μg ml$^{-1}$ rabbit IgG and their differences with different carrier flow stop times (R(50)-3 means that 50 μg ml$^{-1}$ rabbit IgG is employed as sample, and carrier flow is stopped for 3 min). The peak height of 50 μg ml$^{-1}$ rabbit IgG is set at 100% when carrier flow is not stopped.*

between rabbit IgG and protein G, and was used in our further experiments as elution buffer.

*Effects of carrier flow stop time*

The extent of the reaction between substrate and bound HRP-labelled protein A is related to the residence time of substrate in the cartridge. The influence of carrier flow stop time on the peak height is studied for 0 and 50 μg ml$^{-1}$ rabbit IgG using a 1:500 dilution (0.16 U HRP ml$^{-1}$) of HRP-labelled protein A as conjugate. Figure 5 shows the relative peak heights for 0 and 50 μg ml$^{-1}$ rabbit IgG. The peak height of 50 μg ml$^{-1}$ rabbit IgG is set at 100% when the carrier flow has not been stopped. The peak heights for 0 and 50 μg ml$^{-1}$ rabbit IgG increase with the increase of carrier flow stop time, and their differences are linear up to 5 min carrier flow stop time. This result shows that the carrier flow of

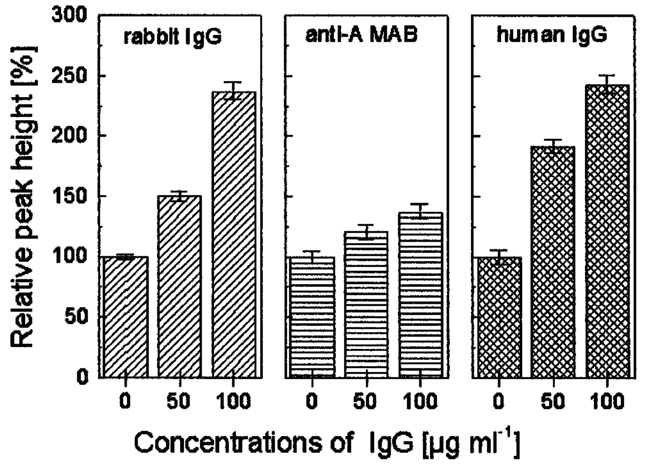

*Figure 6. Relative peak heights for 0, 50 and 100 μg ml$^{-1}$ rabbit IgG, human IgG and anti-A MAB. The peak height of 0 μg ml$^{-1}$ IgG is set at 100%.*

the flow-ELISA system can be stopped in order to increase the sensitivity of the system. However, a compromise between the assay speed and the sensitivity should be made.

*Binding capacity*

A few IgGs, e.g. human IgG and mouse IgG, also bind to protein G. The binding capacity of these IgGs to protein G is compared for 50 μg ml$^{-1}$ and 100 μg ml$^{-1}$ using a 1:200 dilution (0.4 U HRP ml$^{-1}$) of HRP-labelled protein A as conjugate. For the measurement of each IgG, a fresh cartridge packed with protein G-sepharose is also employed. Figure 6 shows the relative peak heights of rabbit IgG, human IgG and anti-A MAB (an IgG-type produced by Boehringer Ingelheim Pharma KG). The peak height of 0 μg ml$^{-1}$ IgG is set at 100% and used as reference. Rabbit IgG and human IgG bind to protein G very well, and the peak height of 100 μg ml$^{-1}$ is about 2.5 times higher than that of 0 μg ml$^{-1}$. However, there is no large difference in peak heights with anti-A MAB, so it has less binding capacity to protein G.

*Reproducibility*

The reproducibility of the system is investigated using 0 and 50 μg ml$^{-1}$ rabbit IgG and a 1:250 dilution (0.32 U HRP ml$^{-1}$) of HRP-labelled protein A. Figure 7 shows the peak heights for 0 and 50 μg ml$^{-1}$ rabbit IgG repeatedly measured. There is a decrease in peak heights for 50 μg ml$^{-1}$ rabbit IgG and a slight increase in peak heights for 0 μg ml$^{-1}$ rabbit IgG. This decay of the binding capacity of protein G-sepharose in the cartridge is caused by the denaturation due to the low pH elution buffer and the substrate solution. However, the capacity decay of the cartridge can be compensated by correcting sample peak heights with reference sample, e.g. 0 μg ml$^{-1}$ rabbit IgG [8]. After the compensation with 0 μg ml$^{-1}$ rabbit IgG, the standard deviation (SD) equals 1.508 and standard error of mean (SEM) equals 0.241 54 for corrected 50 μg ml$^{-1}$ rabbit IgG. The peak heights of samples corrected with 0 μg ml$^{-1}$ rabbit IgG show a good

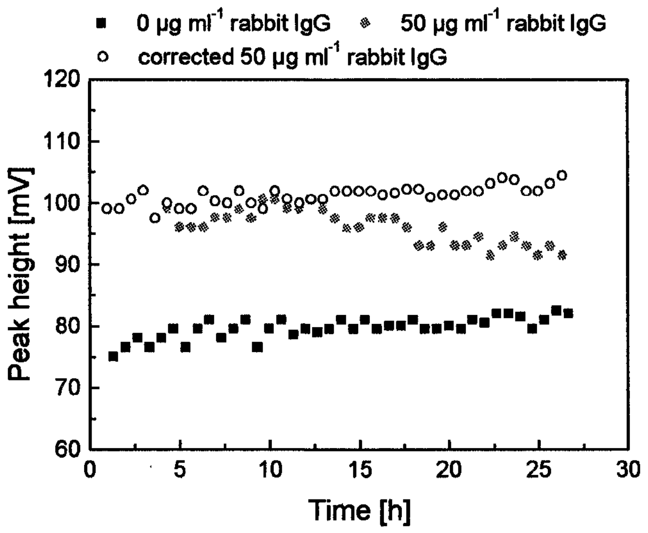

*Figure 7. Peak heights for 0 and 50 μg ml$^{-1}$ rabbit IgG with repeated assay cycle. The peak height of 50 μg ml$^{-1}$ rabbit IgG is corrected on the basis of the peak height difference of the first five samples.*

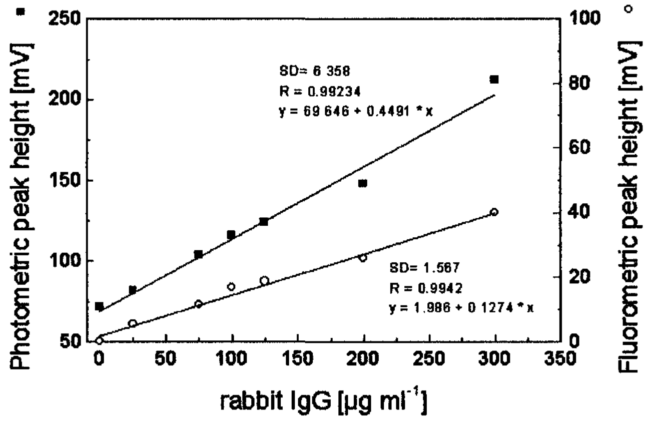

*Figure 8. Calibration curves for rabbit IgG with photometric and fluorometric detection.*

reproducibility of the flow-ELISA system, and that rabbit IgG can be monitored on-line very well, provided that a correction for the decay of a cartridge is adequately performed.

### *Calibration curves*

Figure 8 shows calibration curves for rabbit IgG using a 1:200 dilution (0.40 U HRP ml$^{-1}$) of HRP-labelled protein A. At 0 μg ml$^{-1}$ rabbit IgG there is a photometric detection (about 70 mV) due to the reaction of the unwashed HRP-labelled protein A in the cartridge with a mixture of $H_2O_2$ and ABTS. During the measurment of 300 μg ml$^{-1}$ rabbit IgG, a fluorometric signal of unbound HRP-labelled protein A was also detected. In the elution step, both rabbit IgG and bound HRP-labelled protein A are eluted and detected by a fluorometer. As shown in figure 8, the slope of the calibration curve by the photometric detection is 0.4491 mV (μg ml$^{-1}$)$^{-1}$ in the range of 0 and 300 μg ml$^{-1}$ rabbit IgG, while it is 0.1274 mV (μg ml$^{-1}$)$^{-1}$ with the fluorometric detection. From this result, it can be seen that the flow-injection sandwich ELISA has higher sensitivity and a lower detection limit than the heterogeneous assay.

### Conclusion

A flow-injection sandwich ELISA is described using the model system: protein G, rabbit IgG and HRP-labelled protein A. HRP-labelled protein A bound to rabbit IgG in the cartridge reacts with substrate containing ABTS (redox indicator), and the colour variation of ABTS is detected photometrically. The effects of elution buffers, carrier flow stop time and binding capacity are investigated for the model system. The binding capacity of the cartridge containing protein G-sepharose decreased due to the denaturation caused by the low pH elution buffer and the substrate solution. However, the decay can be corrected to monitor on-line the concentrations of rabbit IgG by compensating the peak height of samples with reference sample. The sensitivity of the flow-injection sandwich ELISA was compared with that of the heterogeneous assay. Even if both rabbit IgG and HRP-labelled protein A were eluted and detected in the heterogeneous immunoassay by a fluorometer, its sensitivity, e.g. slope of calibration curve [0.1274 mV (μg ml$^{-1}$)$^{-1}$] was not higher than that of the flow-injection sandwich ELISA [0.4491 mV (μg ml$^{-1}$)$^{-1}$]. It has been shown in our studies that the flow-injection sandwich ELISA would be an attractive alternative to the on-line monitoring of protein products in biotechnological processes. Further, a flow-injection sandwich ELISA for the on-line monitoring of the rt-PA (recombinant tissue-type plasminogen activator) is envisaged using anti rt-PA as immobilizing material and HRP-labelled anti rt-PA as conjugate.

### Acknowledgment

The authors would like to thank Dr Noe from Boehringer Ingelheim Pharma KG for his help during these studies and for providing anti-A MAB samples.

### References

1. Stöcklein, W. and Schmid, R. D., *Anal. Chim. Acta*, **234** (1990), 83.

2. Brandes, W., Maschke, H. E. and Scheper, Th., *Anal. Chem.*, **65** (1993), 3368.

3. Mattiasson, B. and Hakanson, H., *Advances in Biochemical Engineering and Biotechnology*, Fiechter, A. (ed.) (Heidelberg, Springer) (1992), 81.

4. De.Alwis, W. U. and Wilson, G. S., *Anal. Chem.*, **57** (1985), 2754.

5. De.Alwis, W. U. and Wilson, G. S., *Anal. Chem.*, **59** (1987), 2786.

6. Lee, I. H. and Meyerhoff, M. E., *Mikrochimica Acta*, **111** (1988), 207.

7. Nilsson, M., Hakanson, H. and Mattiasson, B., *Anal. Chim. Acta*, **249** (1991), 163.

8. Nilsson, M., Mattiasson, G. and Mattiasson, B., *J. Biotechnol*, **31** (1993), 381.

9. Beyer, K., Reinecke, M., Noe, W. and Scheper, Th., *Anal. Chim. Acta*, **309** (1995), 301.

10. Rhee, J. I. and Schügerl, K., *Anal. Chim. Acta*, **355** (1997), 55.

